# Salicylic acid: new pathways arising?

**DOI:** 10.3389/fpls.2025.1681791

**Published:** 2025-11-10

**Authors:** Jonas Müller, David Scheuring

**Affiliations:** Plant Pathology, Department of Biology, University of Kaiserslautern-Landau, Landau, Germany

**Keywords:** salicylic acid, vacuole, auxin, NPR (nonexpressor of PR genes), receptor, signaling

## Abstract

While the role of salicylic acid (SA) for plant immunity has been investigated for decades, its function in regulating plant growth and development has only come into focus recently. Several studies indicate that SA – auxin crosstalk plays an important role in mediating SA-induced effects. However, not all findings can be explained by this crosstalk alone and SA-specific effects on intracellular organization have been reported such as inhibition of endocytosis and changes of vacuolar pH and morphology. Notably, several SA-related functions seem to be independent of the SA receptors Nonexpressor of Pathogenesis-Related genes (NPRs). This review summarizes the effects of SA on intracellular organization and predicts the existence of as yet unknown signaling pathways to explain the current findings. We provide a short general introduction including SA biosynthesis and SA signaling and address how NPR-independent intracellular changes necessitate specific signaling to regulate growth and development.

## Introduction

As sessile organisms, plants must constantly adjust their growth and development and adapt to ever-changing biotic and abiotic conditions for survival. Central to this adaptability is a complex network of chemical signaling pathways, with phytohormones at the core (Davies, 2010). Being produced in very small amounts, these low molecular weight compounds have fundamental roles in growth, development, and stress responses (Santner et al., 2009; Bari and Jones, 2009). Through tightly regulated biosynthesis, perception, and signaling, phytohormones maintain homeostasis and support the plant’s developmental plasticity and resilience (Anfang and Shani, 2021).

Historically, five major classes of phytohormones were identified: auxin, cytokinin, abscisic acid, gibberellin, and ethylene. Only in the late 20th century, salicylic acid (SA) was recognized as the sixth phytohormone. Initially studied for its pharmaceutical properties, SA’s importance in plant immunity was first demonstrated in 1979 by R.F. White (White, 1979), who discovered that acetylsalicylic acid, a derivative of SA, could induce pathogen resistance. In the 1980s, further studies revealed SA to be a key inducer of pathogenesis-related (PR) proteins, important defense proteins activated in response to pathogen attack or stress (Antoniw and White, 1980; Loon and Antoniw, 1982; van Huijsduijnen et al., 1986). Later, these findings culminated in the discovery of systemic acquired resistance (SAR) (Métraux et al., 1990), with a breakthrough in the 1990s, when Cao et al. identified NPR1 (Nonexpressor of Pathogenesis-Related genes 1) as central regulator (Cao et al., 1994, 1997). Around 15 years later, it was demonstrated that SA binds directly to NPR3 and NPR4, which in turn controls NPR1 stability (Fu et al., 2012). Nowadays it is well established that SA plays a central role within plant immunity, particularly in mediating defense against biotrophic and hemibiotrophic pathogens (Vlot et al., 2009; Klessig et al., 2018; Ding and Ding, 2020).

Unlike animal cells, each plant cell has the capacity to produce hormones. SA biosynthesis is conserved across plants, bacteria, and fungi, starting with chorismate, a product of the shikimate pathway in the chloroplast. To date, two main pathways have been identified in plants: the isochorismate synthase (ICS) pathway and the phenylalanine ammonia-lyase (PAL) pathway (Lefevere et al., 2020). For the ICS pathway it has been demonstrated that isochorismate is transported from the chloroplast to the cytosol by the Enhanced Disease Susceptibility 5 (EDS5) transporter (Nawrath et al., 2002), where it is eventually converted into SA. Recently, regulation of growth and development has emerged as another important function of SA, extending its role beyond plant immunity.

SA can modulate plant growth in both positive and negative ways, depending on its concentration, exposure duration, and environmental conditions (Rivas-San Vicente and Plasencia, 2011; Huot et al., 2014). In Arabidopsis, SA-deficient mutants like SA induction-deficient 2 (sid2) or *nahG*, expressing a bacterial salicylate hydroxylase gene that degrades SA, typically display enhanced growth (Wildermuth et al., 2001; Friedrich et al., 1995). In line with this, SA-over accumulating mutants such as constitutive expresser of pathogenesis-related genes-5 (*cpr5)* and accelerated cell death 6 (*acd6)* exhibit dwarfism (Bowling et al., 1997; Rate et al., 1999). Together, this highlights an inverse relationship between SA levels and growth. Additionally, SA has been shown to affect cell division and expansion, particularly in roots and leaves, while also playing a role in regulating key developmental transitions such as flowering and senescence (Li et al., 2022). However, the mechanistic basis of most functions related to growth and development is not well understood and evidence emerged that these might be independent from canonical SA signaling.

## Impact of phytohormones on the intracellular organization

**Figure 1:**
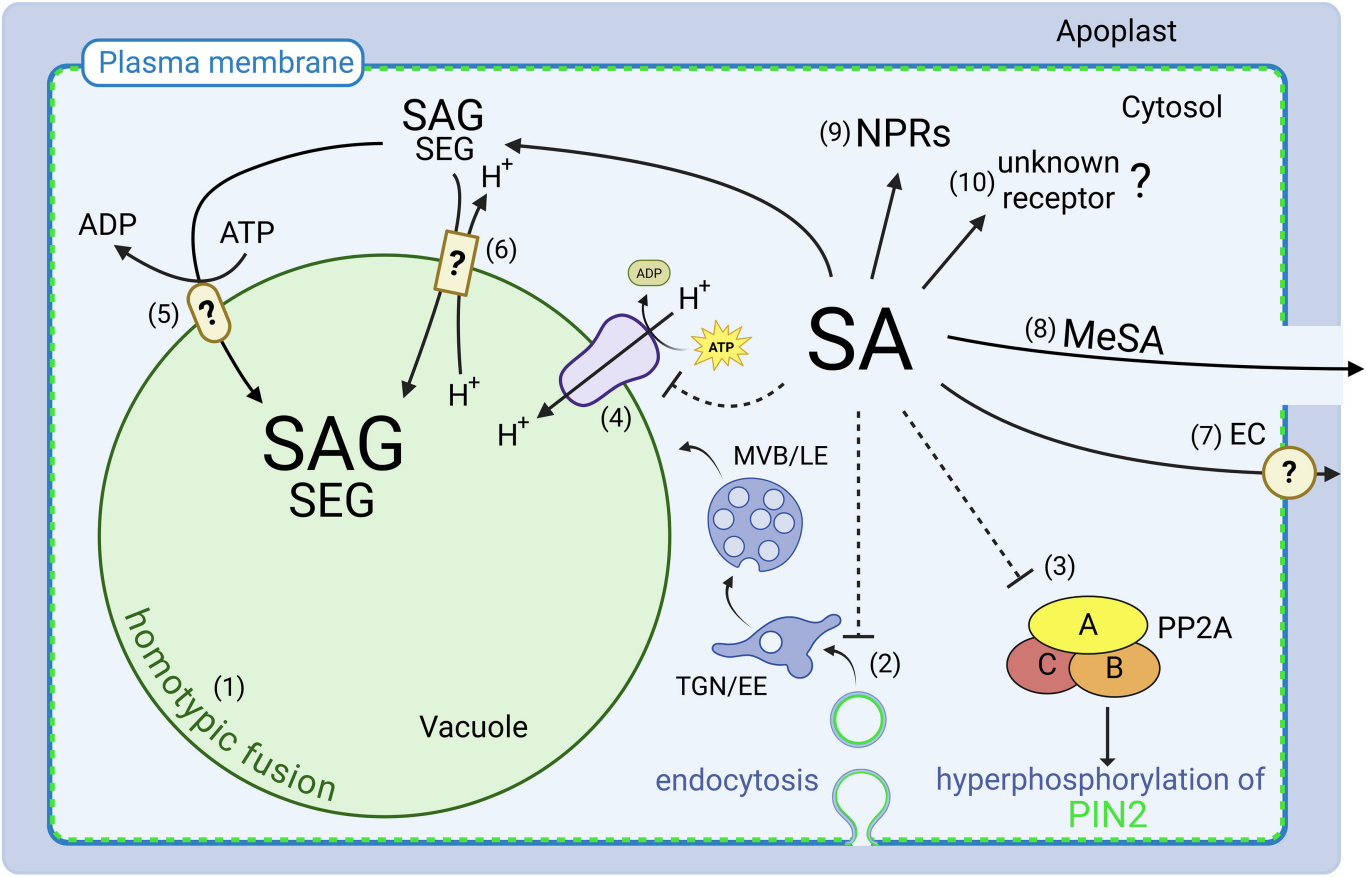
Perception, transport and function of SA. Recent research has expanded the role of SA beyond immunity, but many aspects need to be resolved. (1) SA impacts vacuolar morphology presumably by homotypic fusion. (2) SA inhibits endocytosis, impairing the recycling of key membrane proteins such as the auxin efflux carrier PIN-FORMED 2 (PIN2) (Du et al., 2013). (3) SA interferes with PIN2 polarity by inhibition of the protein phosphatase 2A (PP2A) (Tan et al., 2020). (4) Vacuolar H^+^-ATPase activity is inhibited by SA, resulting in increased vacuolar pH (Müller et al., 2025). (5) Transport of glycosylated SA into the vacuole is dependent on ATP-binding cassette (ABC) transporters (Dean and Mills, 2004) or (6) H^+^-antiport mechanisms (Dean et al., 2005). (7) There is also evidence for the existence of a SA efflux carrier (EC) system (Chen et al., 2001; Clarke et al., 2005; Rocher et al., 2009). (8) Methylation of SA produces the mobile methyl salicylate (MeSA) (Park et al., 2007). (9) While Non-Expressor of Pathogenesis-Related Genes (NPR) proteins have been established as canonical receptors mediating SA’s role in immunity. (10) Emerging evidence suggests that plant growth and development are at least partially independent, implying the existence of as-yet unidentified additional SA receptors. TGN/EE, *Trans*-Golgi Network/Early Endosome; MVB/LE, Multivesicular Body/Late Endosome; SAG, salicylic acid 2-O-β-D-glucoside; TGN/EE = *trans*- Golgi Network/Early Endosome; MVB/LE = Multivesicular Body/Late Endosome; SAG = salicylic acid 2-O-b-D-glucoside; SGE = salicylic acid glucoside ester.

For many phytohormones regulation of growth and development does not only occur via transcriptional changes but on the translational level, often involving changes on the subcellular organization. For auxin, a direct impact on the morphology of the plant’s largest organelle, the vacuole, has been reported (Löfke et al., 2015). Inhibition of cell elongation in the Arabidopsis root was accompanied by limiting vacuole size, indicating that inflation of the vacuole is a prerequisite for plant growth (Scheuring et al., 2016). Thus, a space-filling function was assigned to the vacuole as an energy-saving mechanism to occupy the emerging space in rapidly growing cells (Krüger and Schumacher, 2018; Dünser et al., 2019; Kaiser and Scheuring, 2020; Kaiser et al., 2021). Notably, also SA impacts vacuolar morphology in roots (**Figure 1**), but the relative vacuole size remains unchanged (Müller et al., 2025). Here, not vacuole size but direct inhibition of V-ATPase activity seems to be responsible for limiting cell size and growth. Another mechanism directly affected by auxin and SA is cellular uptake or endocytosis. For auxin, repression of endocytosis has been demonstrated to serve as a means to regulate the abundance of auxin transporters at the plasma membrane (PM) (Paciorek et al., 2005). For SA, a partial inhibition of endocytosis has been reported. Exogenous treatments and endogenously enhanced SA levels both repressed endocytosis of different PM proteins but did not involve the known signaling components (Du et al., 2013). The SA receptor mutants *npr1–1* and *npr1–2 npr3–1 npr4–3* both displayed WT-like behavior, as SA significantly reduced internalization of the auxin efflux carrier PIN-FORMED (PIN1) and PIN2 in all lines. Intriguingly, SA did not affect ligand-induced internalization of the FLAGELLIN SENSING2 (FLS2) receptor which binds peptides of bacterial flagellin (Du et al., 2013). This suggests that the NPR-dependent role of SA within plant immunity and inhibition of (clathrin-mediated) endocytosis are independent mechanisms. In line with this, it was shown that SA and MeSA effects on Arabidopsis pollen tip growth was independent of known NPR3/NPR4 SA receptor-mediated signaling pathways (Rong et al., 2016). SA also inhibited endocytosis in this experimental setup and hardened the evidence for NPR-independent signaling (**Figure 1**). Notably, also other phytohormones impact endocytosis directly. Abscisic acid (ABA) fine-tunes its signaling by regulating the recycling of its own transporters, such as ABCG25, from early endosomes (TGN/EE) to the PM (Park et al., 2016). In addition, ABA induces internalization of other proteins, e.g. the potassium channel KAT1, which contributes to the modulation of stress-related processes through their intracellular recycling (Sutter et al., 2007). Cytokinin also promotes endocytosis by specifically targeting the auxin transporter PIN1, thereby regulating organogenesis (Marhavý et al., 2011). Gibberellins (GAs) have also been shown to modulate PINs by directing them from the vacuolar degradation pathway to the PM and thus change auxin fluxes (Salanenka et al., 2018). Taken together, there are numerous examples that phytohormones, in addition to their transcriptional function, act directly on fundamental cellular processes, including endocytosis and vacuolar trafficking. For SA it has been demonstrated that these functions are at least partially independent of the canonical SA receptors NPRs. Interestingly, many of the observed effects on intracellular organization were involving crosstalk with the key regular for plant growth and development, auxin. In the case of the NPR-independent SA functions, the question arises as to which extent auxin is also involved.

## SA crosstalk with other phytohormones

Many different aspects of growth and development are regulated involving crosstalk between phytohormones. In recent years, considerable progress has been achieved in understanding crosstalk between SA and auxin (Rawat and Laxmi, 2025). SA affects auxin biosynthesis by upregulating Tryptophan Aminotransferase of Arabidopsis 1 (TAA1), a key enzyme in the tryptophan-dependent auxin synthesis pathway as well as Gretchen Hagen 3.5/Weak Ethylene Sensitive 1 (GH3.5/WES1), which is involved in auxin conjugation (Zhang et al., 2007; Pasternak et al., 2019). Moreover, SA has additionally shown to inhibit Catalase 2 (CAT2), decreasing tryptophan accumulation and thus reducing auxin biosynthetic capacity (Ursache et al., 2014; Yuan et al., 2017). SA also negatively influences auxin signaling by interfering with auxin perception via disruption of the auxin receptor TRANSPORT INHIBITOR RESPONSE1 (TIR1) and by stabilizing AUX/IAA proteins, which are transcriptional repressors of auxin-responsive genes (Wang et al., 2007). This results in a global repression of many auxin-inducible genes, e.g. *Small, Auxin-Up RNAs* (*SAURs*), thereby limiting auxin-mediated responses. In 2020, another case of SA-auxin crosstalk was discovered and a parallel SA signaling for plant immunity and growth inhibition proposed (Tan et al., 2020). Here, direct binding of SA to the A subunits of protein phosphatase 2A (PP2A) impact the auxin distribution network. By inhibiting the activity of the PP2A complex, SA prevents dephosphorylation of the auxin transporter PIN2, a target of PP2A. This leads to PIN2 hyperphosphorylation and a loss of polarity (**Figure 1**), impairing auxin transport and auxin-mediated root development. Inhibition of root growth and altered lateral root organogenesis by SA has been reported previously (Pasternak et al., 2019) but Tan et al., demonstrated for the first time that this is independent of NPR-signaling (Tan et al., 2020). Thus, a new SA signaling pathway was proposed which might participate in balancing plant growth and immunity.

However, Müller et al. provided several lines of evidence that crosstalk between SA and auxin might not be sufficient to explain their findings: 1) the auxin receptor triple mutant *tir1 afb2 afb3* and the PIN2 mutant *eir1–4* are fully sensitive to SA-induced root inhibition, 2) quantification of phytohormone levels in seedlings upon SA application did not result in an auxin accumulation and 3) changes of vacuolar morphology upon SA treatment differs significantly from auxin-induced changes (Müller et al., 2025). The specific SA-induced inhibition of V-ATPase activity could be part of another SA signaling pathway, independent of canonical NPR signaling. Together with the NPR-independent inhibition of endocytosis (Rong et al., 2016) and root growth (Tan et al., 2020; Müller et al., 2025), this indicates that other, not yet identified signaling components such as receptors or transporters are involved in SA-mediated growth regulation (**Figure 1**). In some reviews SA is not only described to change auxin distribution but even to antagonize auxin effects (Rawat and Laxmi, 2025; Tian et al., 2025; Bagautdinova et al., 2022). Nonetheless, most of the newer work agrees that the impact of SA on growth repression extends beyond its crosstalk with auxin.

## SA signaling specific for growth and development?

In addition to the canonical SA receptors NPRs, other SA-binding proteins (SABPs), including catalases, glutathione S-transferases and GH3 (acyl acid amido synthetase) have been found, potentially acting as SA receptors (Pokotylo et al., 2019). However, the molecular mechanisms by which most of the SABPs functions in SA signaling remain to be solved. In addition to receptor binding, SA activity can be controlled through chemical modifications, including glycosylation, methylation, and amino acid conjugation (Dempsey and Klessig, 2017). Glycosylation of SA is catalyzed by members of the UDP-glycosyltransferases superfamily, with UGT74F1, UGT74F2 and UGT76B1 being the most abundant in Arabidopsis. UGT74F1 and UGT74F2 preferentially convert SA into its inactive storage form, salicylic acid 2-O-β-D-glucoside (SAG) and salicylic acid glucoside ester (SGE), respectively (George Thompson et al., 2017). UGT76B1 can produce SAG and small amounts of SGE, potentially to finetune SA-mediated immune responses (Zhang et al., 2024). It has been proposed that both, SAG and SGE are transported from the cytosol into the vacuole as inactive storage form of SA (Vaca et al., 2017). Using vacuolar membrane enriched vesicles it was shown, however, that SAG significantly accumulates inside while the majority of SGE was located outside the vacuole (Vaca et al., 2017). In any case, inactivation of SA by modifications reduces levels of active SA. In accordance, overexpression of *UGT76B1* reduces the inhibitory SA effect on root growth while *ugt76b1* knockout mutants show hypersensitivity, likely due to increased levels of non-glycosylated, active SA (Müller et al., 2025). To date, no specific SA transporters at the vacuole have been identified (Anfang and Shani, 2021), although there is evidence for vacuolar import of SA by ATP-Binding Cassette (ABC) transporters and H^+^-antiporters, which might depend on the plant species (Dean and Mills, 2004; Dean et al., 2005; Vaca et al., 2017). Intriguingly, some data indicate the cellular export of SA (Chen et al., 2001; Rocher et al., 2009) although no SA efflux carrier has been identified so far (**Figure 1**).

**Figure 2:**
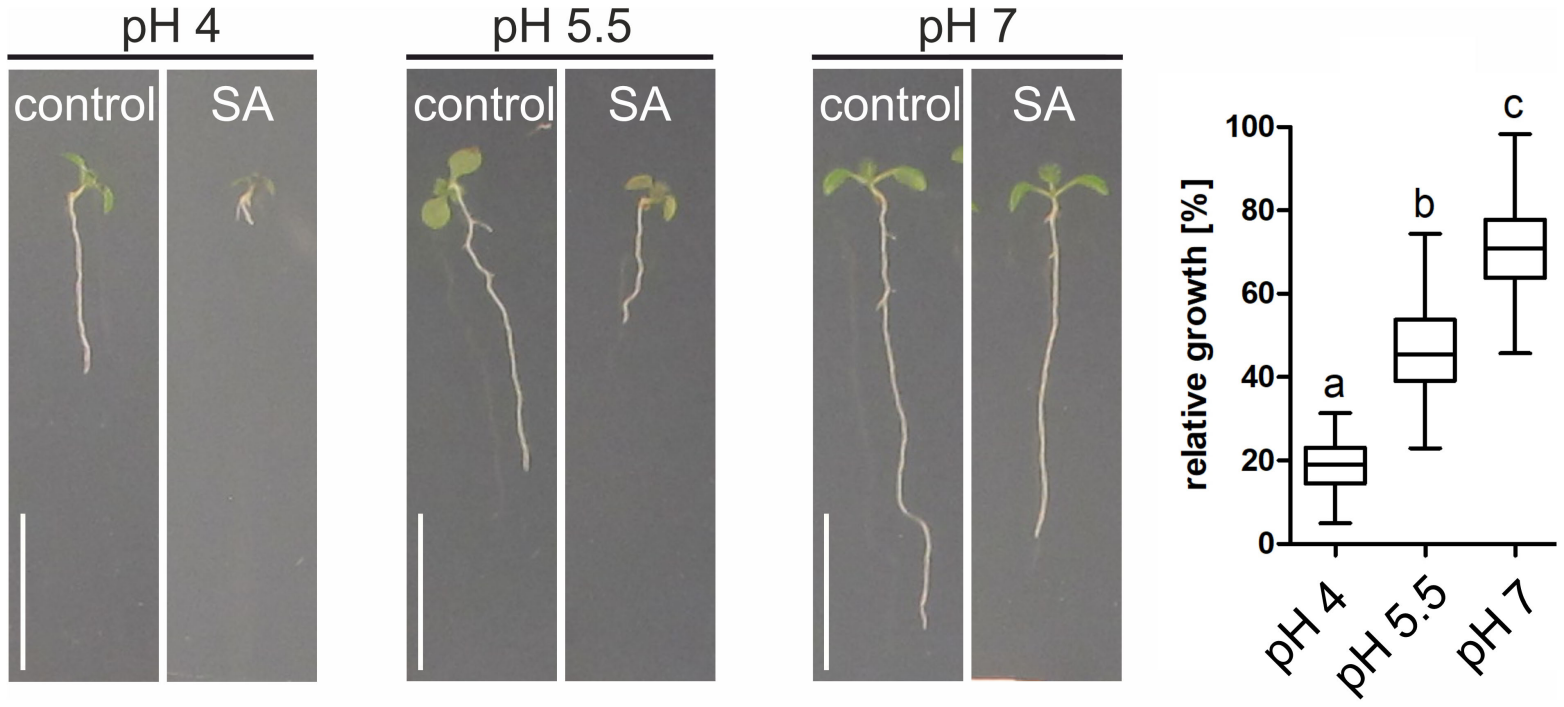
pH-dependent root growth inhibition by salicylic acid. Arabidopsis seedlings were grown for 7 days on media with different pH values. To induce root growth inhibition, 50 µM SA was used. Relative growth was determined by comparing root length from SA-containing media to the average root length of seedlings grown on control plates. Quantification: ANOVA test with Tukey *post-hoc* test. Change in letter equals p ≤ 0.001. Scale bars: 8 mm.

Due to their low molecular weight and chemical properties, most phytohormones can efficiently diffuse across membranes in their protonated (non-polar) form but are deprotonated and trapped because of the neutral pH in the cytosol. According to this ion-trap mechanism, increased SA (pK_a_<3) uptake at low pH conditions can be expected. Indeed, using SA-induced root length inhibition as readout, stronger effects are observed under low pH conditions (**Figure 2**). Still, even at neutral pH root growth is significantly inhibited, suggesting that active transport processes contribute to SA uptake (**Figure 2**, pH 7). In line with this, at neutral pH and hence very little diffusion, SA nevertheless accumulate inside plant cells. Explanations involve a H+ cotransport or a pH-dependent efflux carrier system for SA, but both mechanisms have not been resolved yet (Clarke et al., 2005; Rocher et al., 2006, 2009).

## Conclusion

The role of SA in regulation of plant growth and development has come into focus only recently. Therefore, there are significant gaps in our understanding of perception, signaling and transport in this context. Most intriguingly, several SA processes are independent of the *bona fide* SA receptors NPR, such as inhibition of endocytosis (Rong et al., 2016) and root growth (Tan et al., 2020; Müller et al., 2025). Since multiple SABPs have been identified in addition to the NPRs, it is tempting to speculate that (a) specific receptor(s) exist(s) that mediate SA regulation of plant growth and development. A second intriguing aspect of SA-function is the lack of any specific transporters. Although there is evidence for transport of glycosylated SA into the vacuole (Vaca et al., 2017) and export (excretion) out of the cell (Rocher et al., 2009), no transporter at the tonoplast or the PM has been described to date. This is especially surprising as for other phytohormones several transporters at different compartments and directions have been described (Anfang and Shani, 2021). Taken together, this pleads for the existence of additional SA signaling pathway and transport processes, likely to be discovered in the near (exciting) future.
